# Genomic Profile and Clinical Outcomes in Acute Myeloid Leukemia with Monosomal Karyotype

**DOI:** 10.3390/ijms26125845

**Published:** 2025-06-18

**Authors:** Collins Wangulu, Ehsan Bahrami Hezaveh, Mojgan Zarif, Qianghua Zhou, Winnie Lo, Cuihong Wei, Hassan Sibai, Hong Chang

**Affiliations:** 1 Princess Margaret Cancer Biobank (PMCB), University Health Network, Toronto, ON M5G 2C4, Canada; collins.wangulu@uhn.ca; 2 Department of Laboratory Medicine and Pathobiology, University of Toronto, Toronto, ON M5S 1V4, Canada; qianghua.zhou@uhn.ca (Q.Z.); winnie.lo@uhn.ca (W.L.); cuihong.wei@uhn.ca (C.W.); 3 Department of Laboratory Hematology, University Health Network, Toronto, ON M5G 2C4, Canada; ehsan.bahramihezaveh@uhn.ca (E.B.H.); mojgan.zarif@uhnresearch.ca (M.Z.); 4 Laboratory Medicine Program, Clinical Laboratory Genetics, University Health Network, Toronto, ON M5G 2C4, Canada; 5 Department of Medical Oncology and Hematology, Princess Margaret Cancer Centre, University of Toronto, Toronto, ON M5G 2C4, Canada; hassan.sibai@uhn.ca

**Keywords:** Acute Myeloid Leukemia, monosomal karyotype, mutations, cytogenetics, *TP53* mutation, overall survival

## Abstract

The biology of Monosomal Karyotype Acute Myeloid Leukemia (MK AML) remains unclear, and its mutational profile has not been exclusively assessed. We sought to determine the genomic profile of MK AML patients and its correlation with overall survival (OS). We conducted a retrospective study involving 664 AML patients, identifying 156 (23.5%) with MK AML. The most common monosomies were -17 (41%) and -7 (37%), with 149 (95%) and 138 (88%) having myelodysplasia-related (*MR*) cytogenetics and complex karyotype (*CK*), respectively. Frequent mutations included *TP53* (69%), *DNMT3A* (19%), *TET2* (13%), and *IDH1* (7%). Patients with MK AML with *TP53* mutation (*TP53 Mut*) had shorter OS compared to those with *TP53* wild-type (*WT*) (median OS, 3.9 versus 9.2 months, *p* = 0.002). Our validation study further supports this finding. There was no significant difference in OS related to the presence or absence of CK (*p* = 0.252), *MR* mutations (*p* = 0.252), *DNMT3A* (*p* = 0.264), *TET2* (*p* = 0.264), and *IDH1* (*p* = 0.183) alterations. Co-mutation with novel EPI6 and TAZI signature alterations did not significantly impact OS among MK AML *TP53 Mut* patients, suggesting that *TP53 Mut* remains the dominant driver of outcome in this subgroup. In conclusion, MK AML is a genotypically diverse and high-risk group, with MK AML *TP53 Mut* indicating worse prognosis.

## 1. Introduction

Monosomal Karyotype (MK) is defined by the presence of a single autosomal monosomy, along with at least one additional autosomal monosomy or a structural chromosome abnormality, other than those associated with defining core binding factor (CBF) Acute Myeloid Leukemia (AML) [t (8; 21) (q22; q22), inv (16) (p13.1q22)/t(16; 16) (p13.1; q22)] or acute promyelocytic leukemia [t (15; 17) (q22; q12)]. MK has been observed in 5–10% of newly diagnosed AML patients, increases with age [1,2,3,4], and has no biological sex disparity [5]. These patients have a poor prognosis [2,4,6], showing limited response to conventional chemotherapy [1,2,7] and allogeneic hematopoietic stem cell transplantation (allo-HCT) [1,8], which explains their adverse risk status according to the European LeukemiaNet 2022 (ELN 2022) risk stratification [9].

The cytogenetic profile of MK AML frequently features -5 and -7 as the two most commonly reported monosomies [1,6,10]. Previous studies, however, document various types and frequencies of these lesions [4,6,7]. Other cytogenetic abnormalities associated with MK AML include inv (3) or t (3; 3), abnormal(12p), abnormal(17p), complex karyotype (CK), and myelodysplasia-related (*MR*) cytogenetic changes [4,7,11]. A relatively high frequency of *TP53* mutation (*TP53 Mut*) [12,13] has been reported among MK AML patients, along with underrepresentation of *NPM1* and *FLT3-ITD* mutations [7,12]. Previous MK AML studies have been limited by low patient numbers [5,12], limited access to molecular genomic analysis that restricted targeted mutational profile [5] and lack of an exclusively MK AML patient population [12]. In fact, as far as we know, no previous study has evaluated the mutational profile of exclusively MK AML patients.

Understanding the cytogenetic and molecular profiles of various types of AML helps predict prognosis and guide optimal therapy to enhance outcomes in the evolving field of science [9,14]. Different cytogenetic abnormalities [1,4,7,13,15] and some co-occurring mutations [5,12,16] in MK AML patients have been demonstrated to confer varying clinical outcomes, making them a heterogeneous group that may benefit from characterization based on their clinical outcomes. The poor prognosis of *TP53 Mut* in AML, including MK AML, has been described [17,18,19,20,21,22,23]. However, other mutational changes underlying MK AML and their impact on clinical outcomes are yet to be established.

Our study aims to determine the cytogenetic and mutational profiles of MK AML patients and seeks to correlate these genomic characteristics with overall survival (OS) in a real-world setting.

## 2. Results

### 2.1. Patient Characteristics

We examined 664 patients diagnosed with AML, of whom 156 (23.5%) had MK AML. The median age for patients with MK AML was 71 years (range, 19–94), with the majority (*n* = 82, 52.6%) being male. At the time of diagnosis, they had a median white blood cell (WBC) count of 3.4 (range, 0.1–162.3) × 10^9^ cells/L, hemoglobin levels 8.4 (range, 5.7–14.2) g/dL, platelet count 43 (range, 9–782) × 10^9^ cells/L and BM blast percentage 38.5% (range, 20–95%) (Table 1).

MK AML patients were older compared to their non-MK AML counterparts (*p* = 0.033) and had lower WBC (*p* < 0.001) and platelet (*p* < 0.001) counts. In addition, they showed a greater incidence of CK (*p* < 0.001) and lower rates of allo-HCT (*p* = 0.023) (Table 1).

### 2.2. Genomic Profile

**Figure 1:**
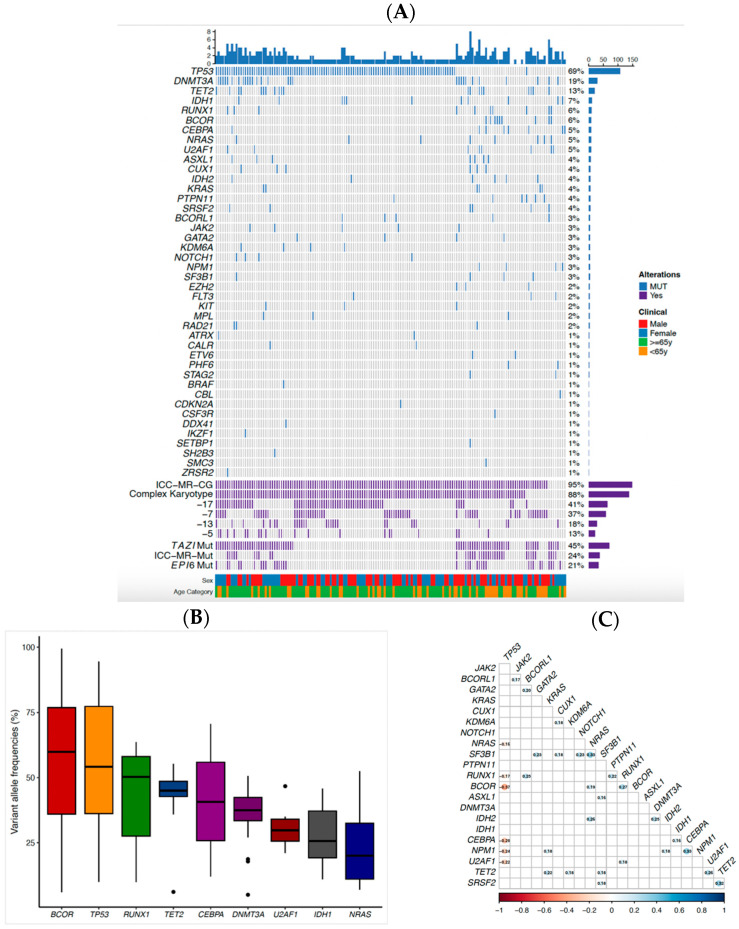
Genomic landscape of the study population. (**A**) Genomic oncoprint of the study cohort. Each column represents a patient, while each row signifies a genomic alteration. The first section is a bar graph showing the number of mutations per patient, followed by the mutations and cytogenetic aberrations examined in the study. The bar plot on the right shows both the percentage and the number of patients with alterations ordered in descending frequency. (**B**) Analysis of variant allele frequencies (VAFs) for the common mutations in the study cohort. The boxplot shows the median (solid line), 25th, 75th percentiles, and minimum and maximum VAF. Values outside the range are indicated as outliers (dots). The order is sorted by median VAF from high to low. (**C**) Correlation plot of the most frequently mutated genes. Blue colour indicates positive correlation and red colour indicates negative correlation. Values represent the coefficients. Only significant pairs are shown.

The most common monosomies observed in the study cohort were -17 (*n* = 64, 41%), -7 (*n* = 58, 37%), -13 (*n* = 29, 18%), and -5 (*n* = 21, 13%) (Figure 1A). In comparison to their non-MK AML counterparts, each of these monosomies was more prevalent in patients with MK AML (Table 1). A total of 149 (95%) and 138 (88%) patients had *MR* cytogenetics and CK, respectively (Figure 1A). *MR* mutations were observed in 37 (24%) patients, whereas two recently described mutation signatures, EPI6 *(CUX1*, *U2AF1*, *EZH2*, *TET2*, *CBL*, or *KRAS)* [24,25] and TAZI *(DNMT3A, SETBP1, CUX1, TET2, ASXL1, BCOR, EZH2, PHF6, RUNX1, SF3B1, SRSF2, STAG2, U2AF1 or ZRSR2)* [25,26] were seen in 33 (21%) and 71 (45%) patients, respectively.

Forty-two (77.8%) of the 54 individual genes targeted for analysis were mutated in 150 (96.2%) MK AML patients. A total of 313 mutations were present, with a median of 2 and a range of 0 to 8 mutations per patient across the 42 genes. Nine genes were mutated in more than 5% of the patients, including *TP53 Mut* (*n* = 108, 69%), *DNMT3A* (*n* = 29, 19%), *TET2* (*n* = 20, 13%), *IDH1* (*n* = 11, 7%), *RUNX1* (*n* = 10, 6%), *BCOR* (*n* = 9, 6%), *CEBPA* (*n* = 8, 5%), *NRAS* (*n* = 8, 5%), and *U2AF1* (*n* = 8, 5%) as illustrated in Figure 1A. Among these genes, *BCOR*, *TP53*, and *RUNX1* had a median variant allele frequency (VAF) greater than 50% (Figure 1B). Thirty-one pairs of gene mutations demonstrated a significant correlation. We identified a significant negative correlation between the *TP53 Mut* and six genes: *NRAS, RUNX1, BCOR, CEBPA, NPM1* and *U2AF1*. In addition, *NPM1—CEBPA*, *SF3B1—NRAS* and *SRSF2* -*TET2* exhibited the strongest positive correlations among these patients (Figure 1C).

### 2.3. Overall Survival (OS) and Impact of Allo-HCT on MK AML Patients

**Figure 2:**
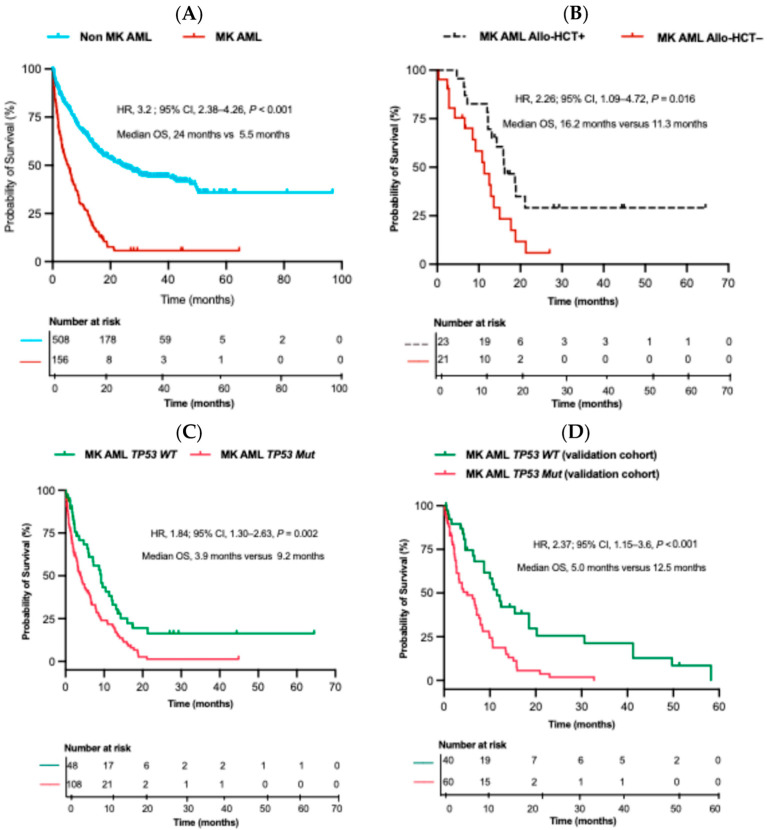
Kaplan–Meier survival plots of OS of the study and validation cohorts. (**A**) Kaplan–Meier plot of OS based on the presence or absence of MK among all AML patients. (**B**) Kaplan–Meier plot of OS based on allogeneic hematopoietic transplant (allo-HCT) status of AML MK patients in the study. (**C**) Kaplan–Meier plot of OS based on *TP53* mutation (*TP53 Mut*) status of MK AML patients in the study. (**D**) Kaplan–Meier plot of OS based on *TP53 Mut* of the validation cohort.

MK AML patients had significantly shorter OS compared to their non-MK AML counterparts (median OS, 5.5 months versus 24 months, *p* < 0.001) (Figure 2A). Regarding their treatment, 47 (30.1%) MK AML patients received only supportive therapy. Meanwhile, 66 (42.3%) and 43 (27.6%) underwent intensive and low-intensive induction chemotherapy, leading to 30 (45.4%) and 9 (20.9%) achieving CR after the first cycle of induction therapy, respectively. In total, 23 (14.7%) patients received allo-HCT while in CR following different cycles of chemotherapy. In contrast, 21 (13.7%) who also reached CR during different cycles did not receive transplantation (Table A2). Overall, recipients of allo-HCT had significantly better OS compared to those who reached CR but were not transplanted (median OS, 16.2 months versus 11.3 months, *p* = 0.016) (Figure 2B).

### 2.4. Impact of TP53 Mutation on OS

We categorized MK AML patients in our study based on their *TP53 Mut* status. Among all MK AML patients in the study, 108 (69.2%) had *TP53 Mut* (MK AML *TP53 Mut*), while 48 (30.8%) were MK AML *TP53* wild-type (MK AML *TP53 WT*). MK AML *TP53 Mut* patients were older (*p* = 0.008) and had a higher frequency of CK (*p* < 0.001) compared to their MK AML *TP53 WT* counterparts. In addition, fewer of these patients underwent allogeneic transplantation (allo-HCT) (*p* = 0.004) (Table 2). Kaplan–Meier analysis showed significantly shorter OS among MK AML *TP53 Mut* compared to the MK AML *TP53 WT* group in the study (median OS, 3.9 versus 9.2 months, *p* = 0.002) (Figure 2C).

We separately analyzed how the presence or absence of CK, monosomies, ICC *MR* mutations, and the most frequent mutations within our study cohort affect OS. Our findings indicated inferior OS related to the presence of monosomy 17 (*p* = 0.009). We found no significant difference in OS related to the presence or absence of CK (*p* = 0.252), ICC *MR* mutations (*p* = 0.252), *DNMT3A* (*p* = 0.264), *TET2* (*p* = 0.264), and *IDH1* (*p* = 0.183) among MK AML patients (Table 3).

We further conducted Cox proportional hazards regression analysis, incorporating covariates known to affect outcomes in AML. We excluded complex karyotype (CK) from our analysis due to its overrepresentation in MK AML [5,7,27]. Our study comprised 138 (88.5%) MK AML patients with CK (Table 1). In addition, CK is also strongly associated with *TP53 Mut* [19,28,29] and did not show significance in the univariate analysis. Our multivariate analysis revealed that allo-HCT (*p* < *0*.001), low-intensity (*p* < *0*.001), and intensive induction chemotherapy (*p* < *0*.001) significantly lowered the risk of death among MK AML patients. Conversely, the presence of *TP53 Mut* was associated with a higher risk of mortality. Age and WBC count showed no significant correlation (Table 4).

### 2.5. Impact of Co-Alterations on MK AML in TP53 Mut and WT Patients

**Figure A1:**
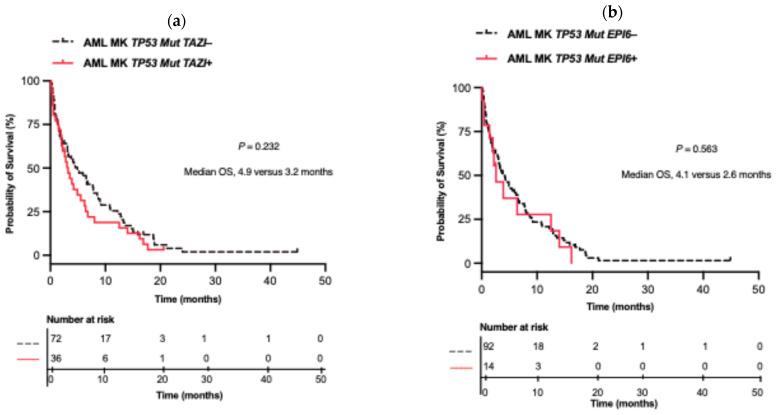
Kaplan–Meier survival plots of OS among AML MK *TP53 Mut* patients based on their *TAZI* and *EPI6* signature mutation status. (**a**) Kaplan–Meier survival plots of OS among AML MK *TP53 Mut* patients based on their *TAZI* signature mutation status. (**b**) Kaplan–Meier survival plots of OS among AML MK *TP53 Mut* patients based on their *EPI6* signature mutation status.

Two co-mutation signatures, *EPI6* [25] and *TAZI* [25,26], have recently been identified and shown to predict adverse clinical outcomes among *TP53 Mut* patients with myeloid neoplasia (MN) [25]. In our study population, there was no difference in OS among MK *TP53 Mut* patients when categorized based on the presence or absence of *TAZI* (*p* = 0.232) (Figure A1a) and *EPI6* (*p* = 0.563) mutation signatures (Figure A1b). In addition, no difference in OS was observed among the AML MK *TP53 WT* group based on the presence or absence of CK (*p* = 0.771), *MR* cytogenetics (*p* = 0.135) and *MR* mutations (*p* = 0.922) and (Table A1).

### 2.6. Validation Cohort

To validate our findings, we utilized a publicly available dataset from the BEAT AML 2.0 study [30]. We analyzed 100 AML MK patients with a median age of 65 (21–83) years, whose clinical and genetic analysis data were accessible. The distribution of biological sex (*p* = 0.394) and *TP53 Mut* status (*p* = 0.129) was comparable to our cohort, although they represented a relatively younger (*p* = 0.002) population (Table A3).

Similar to the approach used in our study population, we categorized them according to their *TP53 Mut* status, with 60 (60%) being AML MK *TP53 Mut* and 40 (40%) AML MK *TP53 WT*. Kaplan–Meier analysis of OS among AML MK *TP53 Mut* patients and their MK *TP53 WT* counterparts demonstrated a significant difference in OS (median OS of 5 versus 12.5 months, *p* < 0.001) (Figure 2D).

## 3. Discussion

Describing the genomic landscape of the heterogeneous MK AML patient group undoubtedly enhances our understanding of the disease biology, potential prognostic classification, and the development of treatment strategies in this era of precision medicine and highly personalized care. MK AML patients in our study cohort represented 23.5% of all AML patients, which is comparable to those described previously [1,7], especially considering our older study population (Table 1). Similar to earlier studies, they were older [2,4,7] with associated lower WBC counts [5,6,12] as compared to their non-MK AML counterparts. We describe their genomic profile in the context of the few existing studies [5,12] and our correlation with clinical outcomes identifies an ultra-adverse risk subgroup based on their OS in a real-world setting.

Our study identifies -17 and -7 as the two most frequent monosomies, similar to Weinberg et al. [5], which contrasts other studies that found -5 and -7 as the most frequent [2,6,10]. Variations in these frequencies can be attributed to differences in study geographical settings [1]. Most importantly, it has previously been established that there is no difference in prognosis resulting from the presence of any specific monosomy within the context of the MK AML [1,4]. Monosomy 17 is part of the spectrum of abnormalities linked to the loss of function or deletion of the *TP53* gene found on chromosome 17p13.1 [31]. In our study, 58 (90.6%) MK AML patients with monosomy 17 had *TP53 Mut*, which supports our findings of an inferior survival among these patients (Table 3). Co-occurrence of unfavourable cytogenetics, such as CK and *MR* cytogenetic abnormalities in MK AML is not uncommon [4,13] and has been shown to worsen clinical outcomes [7]. In our cohort, 88.5% and 95% of MK AML patients had CK and MR cytogenetic abnormalities, respectively, which is comparable to other studies [4,10,32] and could partly explain the overall poor prognosis in this group of patients.

The association and adverse impact of *TP53 Mut* on MK AML has been previously described [1,13], along with its relatively high prevalence in a subset of CK MK patients [12]. With a frequency of 69%, *TP53 Mut* was the most common mutation in our study cohort. Indeed, the ineffective DNA repair mechanisms and impaired apoptosis induced by *TP53 Mut* in leukemic cells has been suggested as the cause of the karyotype complexity and adverse chromosomal alterations observed in *TP53 Mut* AML [17,18]. This is in addition to being a potential mechanism for MK AML and the presence of co-occurring adverse cytogenetics among these patients [1]. Our study also identifies *DNMT3A* (19%), *TET2* (13%), *IDH1* (7%), and *RUNX1* (6%) as the other most frequent altered genes in this group of patients. Leung et al. found *RUNX1* (18%), *DNMT3A* (16%), *BCOR* (14%), *NRAS* (13%) as the most frequent mutations among MK AML patients with CK [12]. Differences in the frequency and types of mutations are likely due to their specific cohort consisting exclusively of CK MK AML patients. MK AML has been found to have a lower representation of *NPM1* and *CEBPA* mutations [5], which is similar to the findings in our study and potentially explains the adverse clinical outcomes observed in this group of patients.

We identify MK AML *TP53 Mut* as a distinct entity associated with poorer clinical outcomes within the heterogeneous MK AML group. Compared to their MK AML *TP53 WT* counterparts, they are older and have a significantly shorter OS (*p* = 0.002). We observed similarly worse OS among this group of patients in our validation dataset from the Beat AML 2.0 cohort [30], which represents a robust, contemporary, and multicenter dataset. In addition, there was no significant difference in OS related to the presence or absence of CK (*p* = 0.252), ICC *MR* mutations (*p* = 0.252), *DNMT3A* (*p* = 0.264), *TET2* (*p* = 0.264), and *IDH1* (*p* = 0.183) among AML MK patients. *TP53 Mut* also maintained its independent prognostic significance in our Cox proportional hazards regression analysis that included covariates known to affect clinical outcomes in AML (Table 4). Other studies indicate a poorer prognosis for MK AML when there is an additional structural chromosomal abnormality or monosomy [1,4] or the presence of CK [13]. However, *TP53 Mut* in AML strongly explains the underlying genetic predisposition to these frequently co-occurring adverse cytogenetic abnormalities [33,34].

Recently, two novel co-mutation signatures, *EPI6* mutations that comprise six genes [24,25] and a 14-gene Tazi signature mutation [25,26] have been shown to have predictive ability for inferior clinical outcomes among *TP53 Mut* patients with Myeloid Neoplasia (MN) [24,25,26]. Considering the previously observed high incidence of *TP53 Mut* among MK AML patients [12], we assessed these signature mutations in our *TP53 Mut* MK AML cohort, as no studies, to the best of our knowledge, have been conducted to determine their incidence and prognostic utility among these patients. There was no difference in OS among *TP53 Mut* MK AML patients based on the presence or absence of these two signature mutations in our study. Potential reasons could be due to the fact that *TAZI* signature was developed outside the context of *TP53 Mut* AML [26], whereas *EPI6* was specifically designed within a *TP53 Mut* MN cohort [24]. Additionally, the two signature mutations share overlapping genes. This finding, however, further suggests that *TP53 Mut* remains the dominant driver of outcome among *TP53 Mut* MK AML patients in the context of OS.

Our study cohort comprised a relatively large number of MK AML patients across a wide age range who received either intensive or non-intensive treatment that reflects the “real-world” patient scenario. However, the single-centre nature of our study limits the generalizability of its findings to other settings. Regional treatment protocols or referral bias may also influence outcomes. Notably, treatment protocols in this setting are more uniform than in multicenter studies. Lastly, we used OS as the primary predicted outcome, which is comparable with other studies, but it is acknowledged that patient mortality may result from treatment toxicity or other causes unrelated to leukemic biology itself. Future multicenter prospective studies with larger patient cohorts of MK AML are needed to confirm our data and comprehensively evaluate our proposed genomic categorization of MK AML patients.

## 4. Materials and Methods

### 4.1. Patients and Samples

We carried out a single-centre retrospective cohort study focusing on MK AML patients. These were selected from a group of AML patients diagnosed from 2016 to 2022, all of whom had comprehensive clinical and diagnostic data, including cytogenetic and molecular testing. The AML diagnoses were reviewed and reclassified according to the 2022 International Consensus Classification (ICC) and subsequently categorized as MK AML based on the cytogenetic results. Furthermore, we utilized a publicly available dataset from the BEAT-AML 2.0 study [30] to validate our findings on defining a worse outcome category. This study received approval from the University Health Network (UHN) Research Ethics Board, and the study procedures adhered to the principles outlined in the Declaration of Helsinki. Patients in the study underwent baseline laboratory testing at diagnosis for Complete Blood Count, bone marrow (BM) blast percentage as well as conventional cytogenetics and molecular testing in accordance with established guidelines. Clinical data, encompassing the age at diagnosis, biological sex, administered chemotherapy, allogeneic hematopoietic stem cell transplantation (allo-HCT) status, conventional karyotype analysis, next-generation sequencing (NGS) results, and survival status, were collected. Individuals with incomplete clinical and laboratory data were excluded from the study.

### 4.2. Treatment

Patients eligible for intensive induction chemotherapy (IC) received regimens such as 7 + 3 (cytarabine plus daunorubicin), 7 + 3 plus midostaurin for *FLT3*-mutated AML or FLAG-IDA (fludarabine, cytarabine, filgrastim, and idarubicin). Those who were ineligible received low-intensity therapy (LIT) regimens, which included hypomethylating agents (azacitidine) alone or in conjunction with the BCL2 inhibitor (venetoclax). Patients with intermediate and high-risk disease in their first complete remission (CR) underwent allo-HCT, as did those with refractory or relapsed disease who achieved remission after salvage chemotherapy, provided an available donor was identified. The best supportive care was provided to patients deemed unsuitable for IC or LIT.

### 4.3. Cytogenetic Analysis

Cytogenetic analysis was performed on diagnostic bone marrow samples using standard chromosome banding techniques, and nomenclatures were provided following the International System for Human Cytogenomic Nomenclature (ISCN) recommendations [35]. Each patient was evaluated for a minimum of 20 metaphases. Monosomal and complex karyotypes were identified in accordance with the European LeukemiaNet (ELN) guidelines.

### 4.4. Molecular Testing

Molecular analysis utilized total cellular DNA extracted from samples of peripheral blood and BM. In patients diagnosed prior to 2018, targeted sequencing (TAR-SEQ) was conducted using a 54-gene next-generation sequencing (NGS) myeloid panel [36]. NGS was performed in patients diagnosed after 2018 using a custom hybrid-capture–based myeloid panel consisting of 49 genes [37]. The detection limit for variant calling was set at 2%, and only pathogenic mutations were considered. Variants of uncertain significance were excluded from the analysis.

### 4.5. Statistical Analysis

Categorical variables were expressed as counts and percentages, while continuous variables were summarized using median and range. Association between variables was assessed using Pearson’s chi-square test and Fisher’s exact test for categorical variables, as well as the Kruskal–Wallis test and Mann–Whitney U test for continuous variables. OS was measured from the initial diagnosis to death from any cause or the last follow-up and analyzed by the Kaplan–Meier method. Differences in OS among the risk groups were established using log-rank test. Pairwise comparison of survival between two groups was also performed using log-rank test reporting the statistical significance, hazard ratios (HRs) and 95% confidence intervals (CIs). Multiple comparisons were controlled for false discovery using the Benjamini–Hochberg procedure to adjust the *p* values [38]. We conducted a multivariable Cox proportional hazards regression analysis that included covariates known to affect clinical outcomes in AML. *p* values <0.05 were considered statistically significant. Statistical analyses were executed using R software version 4.4.1, and GraphPad Prism software version 10.3.1.

## 5. Conclusions

In conclusion, MK AML represents a genotypically and clinically diverse group of patients in the ELN 2022 adverse risk category. MK AML *TP53 Mut* indicates worse outcomes under the current standard of care.

## Figures and Tables

**Table 1:** Demographic and clinical characteristics of the study cohort.

Variable	AML (*n* = 664)	MK − AML (*n* = 508)	MK + AML (*n* = 156)	MK − AML vs. MK + AML
Male:Female Sex	377:287	295:213	82:74	0.231
Age (y), median [range]	69 [18–95]	69 [18–95]	71 [19–94]	0.033 ^a^
WBC Count × 10^9^/L, median [range]	5.9 [0.1–328.7]	8.0 [0.3–328.7]	3.4 [0.1–162.3]	<0.001 ^a^
Hemoglobin, g/dL, median [range]	8.6 [3.7–19.7]	8.6 [3.7–19.7]	8.4 [5.7–14.2]	0.598 ^a^
Platelet × 10^9^/L, median [range]	55 [9.0–2726]	64 [10–2726]	43 [9–782]	<0.001 ^a^
BM Blast percentage, *n* (%)	45 (13–97)	48 (13–97)	38.5 (20–95)	0.002 ^a^
Complex Karyotype, *n* (%)	192 (28.9%)	54 (10.6%)	138 (88.5%)	<0.001 ^b^
Monosomy 7	76 (48.7%)	18 (3.5%)	58 (37.2%)	<0.001 ^b^
Monosomy 17	64 (9.6%)	0	64 (41%)	<0.001 ^b^
Monosomy 13	28 (4.2%)	0	28 (17.9%)	<0.001 ^b^
Monosomy 5	21 (3.2%)	0	21 (13.5%)	<0.001 ^b^
Transplant, *n* (%)	141 (21.2%)	118 (23.2%)	23 (14.7%)	0.023 ^b^

Abbreviations: AML, Acute Myeloid Leukemia; MK, monosomal karyotype; WBC, white blood cell; BM, bone marrow. ^a^—Mann–Whitney *U* test. ^b^—Pearson’s Chi-Square test.

**Table 2:** Clinical characteristics of MK AML patients based on their *TP53* mutation status.

Variable	MK + AML *TP53* + (*n* = 108)	MK + AML *TP53* − (*n* = 48)	MK + AML *TP53 +* vs. MK *+* AML *TP53 −*
Male:Female Sex	53:55	29:19	0.190 ^a^
Age (y), median [range]	73 [36–94]	65 [19–91]	0.008 ^a^
WBC Count × 10^9^/L, median [range]	3.1 [0.1–76.9]	3.8 [0.1–162.3]	0.184 ^a^
Hemoglobin, g/dL, median [range]	8.3 [5.7–12.3]	8.9 [6.4–14.2]	0.018 ^a^
Platelet × 10^9^/L, median [range]	42 [9–782]	44 [9–408]	0.586 ^a^
BM Blast percentage, *n* (%)	35 (20–91)	46 (20−95)	0.070 ^a^
Complex Karyotype, *n* (%)	107 (99.1%)	31 (64.6%)	<0.001 ^b^
Transplant, *n* (%)	10 (9.3%)	13(27.1%)	0.004 ^b^

Abbreviations: AML, Acute Myeloid Leukemia; MK, monosomal karyotype; WBC, white blood cell; BM, bone marrow. ^a^—Mann–Whitney *U* test. ^b^—Pearson’s Chi-Square test.

**Table 3:** Analysis of overall survival in AML MK patients based on their frequent genomic alterations, excluding *TP53* mutation.

Variable		MK AML (*n* = 156)	Median OS (Months)	HR	95% CI	*p* Value
CK	Present	138 (88.5%)	4.9	0.64	0.39–1.05	0.252
	Absent	18 (11.5%)	9.2			
Monosomy 17	Present	64 (41.0%)	3.3	1.77	1.22–2.57	0.009
	Absent	92 (59.0%)	7.9			
Monosomy 7	Present	58 (37.2%)	4.9	1.30	0.91–1.88	0.382
	Absent	98 (62.8%)	6.6			
Monosomy 13	Present	29 (18.6%)	3.3	1.20	0.73–1.95	0.439
	Absent	127 (81.4%)	5.9			
Monosomy 5	Present	21 (13.5%)	3.5	1.55	0.90−2.69	0.183
	Absent	135 (86.5%)	6.0			
ICC *MR* Mutations	Present	37 (23.7%)	7.2	1.36	0.93–1.99	0.252
	Absent	119 (76.3%)	4.7			
*DNMT3A*	Present	29 (18.6%)	3.5	0.76	0.47–1.22	0.264
	Absent	127 (81.4%)	5.9			
*TET2*	Present	20 (12.8%)	2.3	0.72	0.41–1.27	0.264
	Absent	136 (87.2%)	5.9			
*IDH1*	Present	11 (7.1%)	8.7	1.88	1.11−3.18	0.183
	Absent	145 (92.9%)	5.2			

NOTE: The table presents a summary of findings from the Kaplan–Meier analysis of Overall Survival (OS) for study patients based on the presence or absence of Complex Karyotype (CK), Monosomies, *MR* (myelodysplasia-related mutations) as defined by the International Consensus Classification), and the most frequent mutations observed in the study cohort. The median overall survival (OS), hazard ratio (HR), 95% confidence interval (CI), and *p*-values are reported. To control for false discovery in multiple comparisons, the Benjamini–Hochberg procedure was used for adjusting the *p* values.

**Table 4:** Cox proportional hazards regression analysis of variables among MK AML patients.

Variable		Hazard Ratio	95% CI	*p* Value
Age [Years]		1.01	0.66–1.54	0.965
WBC Count		1.00	0.99–1.01	0.949
*TP53 Mut*		1.61	1.06–2.46	0.026
Induction Chemotherapy	Low-Intensity	0.25	0.16–0.41	<0.001
	Intensive	0.32	0.2–0.52	<0.001
Allo-HCT		0.25	0.14–0.47	<0.001

Abbreviations: Allo-HCT, Allogeneic hematopoietic stem cell transplantation; CI, confidence interval; *TP53 Mut*, TP53 mutation; WBC, white blood cell.

## Data Availability

The data presented in this study are available on request from the corresponding author.

## Abbreviations

The following abbreviations are used in this manuscript:

Allo-HCT	Allogeneic hematopoietic stem cell transplantation
AML	Acute Myeloid Leukemia
BM	Bone marrow
CK	Complex karyotype
CR	Complete remission
ELN 2022	European LeukemiaNet 2022
FLAG-IDA	Fludarabine, Cytarabine, Filgrastim, and Idarubicin
IC	Induction chemotherapy
ICC	International Consensus Classification
ISCN	International System for Human Cytogenomic Nomenclature
LIT	Low-intensity therapy
MK	Monosomal Karyotype
MK AML	Acute Myeloid Leukemia with Monosomal Karyotype
MN	Myeloid Neoplasia
*MR*	Myelodysplasia-Related
NGS	Next-generation sequencing
NK	Normal karyotype
Nove-HiDAC	Mitoxantrone, Etoposide, and modified high-dose cytarabine)
OS	Overall survival
SPSS	IBM Statistical Package for the Social Sciences
TAR-SEQ	Targeted sequencing
*TP53 Mut*	*TP53* mutation
UHN	University Health Network
VAF	Variant allele frequency
WBC	White blood cell
*WT*	Wild-type

## Appendix A

**Table A1:** Analysis of overall survival in AML MK *TP53 WT* patients based on complex karyotype (CK), myelodysplasia-related (*MR*) mutations, and *MR* cytogenetics.

Variable		MK AML *TP53 WT* (*n* = 48)	Median OS (Months)	HR	95% CI	*p* Value
CK	Present	31 (64.5%)	7.1	1.112	0.55–2.25	0.771
	Absent	17 (35.4%)	9.2			
*MR* Cytogenetics	Present	40 (83.3%)	7.2	0.419	0.177–1.99	0.135
	Absent	8 (16.7%)	9.2			
*MR* mutations	Present	26 (54.1%)	9.2	1.034	0.53–2.03	0.922
	Absent	22 (45.8%)	8.7			

NOTE: The table presents a summary of findings from the Kaplan–Meier analysis of Overall Survival (OS) for study patients.

The table is based on the presence or absence of Complex Karyotype (CK), *MR* (myelodysplasia-related mutations) as defined by the International Consensus Classification, and *MR* cytogenetics observed among AML MK *TP53 WT* patients in the study. The median OS, hazard ratio (HR), 95% confidence interval (CI), and *p*-values are all provided.

**Table A2:** Treatment profile of patients in the study.

Variable		MK + (*n* = 156)	CR	MK + *TP53* + (*n* = 108)	MK + *TP53* − (*n* = 48)
Induction Chemotherapy	Supportive	47 (30.1%)		33 (30.6%)	14 (29.2%)
	Intensive	66 (42.3%)	30 (45.4%) ^a^	43 (39.8%)	23 (47.9%)
	Less Intensive	43 (27.6%)	9 (20.9%) ^a^	32 (29.6%)	11 (22.9%)
Allo-HCT	Transplanted	23 (14.7%)	23 (14.7%) ^b^	10 (9.3%)	13 (27.1%)
	Not Transplanted	133 (85.3%)	21 (13.7%) ^b^	14 (13%)	7 (14.6%)

Abbreviations: MK, monosomal karyotype; CR, complete remission; Allo-HCT, allogeneic hematopoietic transplantation. ^a^—CR after the first cycle of induction chemotherapy, ^b^—CR during different cycles of chemotherapy.

**Table A3:** Comparison of baseline characteristics between the study and validation cohorts.

Variable		Study Cohort (*n* = 156)	Validation Cohort (*n* = 100)	*p*-Value
Age (years)	Median (min-max)	71 (19–94)	65 (21–83)	0.002 ^a^
Sex	Male Female	82 (52.6%) 74 (47.4%)	58(52%) 42 (42%)	0.394 ^b^
*TP53 Mut* Status	AML MK *TP53 Mut*	108 (69%)	60 (60%)	
	AML MK *TP53 WT*	48 (31%)	40 (40%)	0.129 ^b^
Transplant Status	Allo-HCT	23 (14.7%)	21 (21%)	0.196 ^b^

Abbreviations: AML, Acute Myeloid Leukemia; MK, monosomal karyotype; *TP53 Mut*, mutated *TP53* gene; allo-HCT, allogeneic hematopoietic transplantation. ^a^—Mann–Whitney *U* Test, ^b^—Pearson’s Chi Square.
